# Beneficial use of hyperbaric process conditions on the welding of high-strength low alloy steels

**DOI:** 10.1038/s41598-022-16184-5

**Published:** 2022-07-20

**Authors:** K. Treutler, S. Brechelt, H. Wiche, V. Wesling

**Affiliations:** 1 Clausthal Centre of Materials Technology - University of Technology Clausthal, Leibnizstr. 9, 38678 Clausthal-Zellerfeld, Germany; 2 Institute of Welding and Machining - University of Technology Clausthal, Agricolastr. 2, 38678 Clausthal-Zellerfeld, Germany

**Keywords:** Engineering, Materials science, Metals and alloys

## Abstract

Hyperbaric welding is used for different steels in many underwater applications as a repair welding process. A difference between wet and dry welding processes can be made. Due to the increased ambient pressure, these processes have special features inherent in the process that influence the cooling and penetration behaviour. The positive use of these effects outside underwater applications is currently rarely addressed in science and application. The presented work establishes these advantages on the basis of a higher strength structural steel and characterizes the effects on the microstructure of a joined S700MC steel and on the mechanical properties of the joint. It will be shown that a hyperbaric environment can be used to orient the weld more towards the depth of the sheet. Furthermore, it will be shown that this change leads to modified cooling, which in itself influences the mechanical properties of the weld seam.

## Introduction

**Figure 1:**
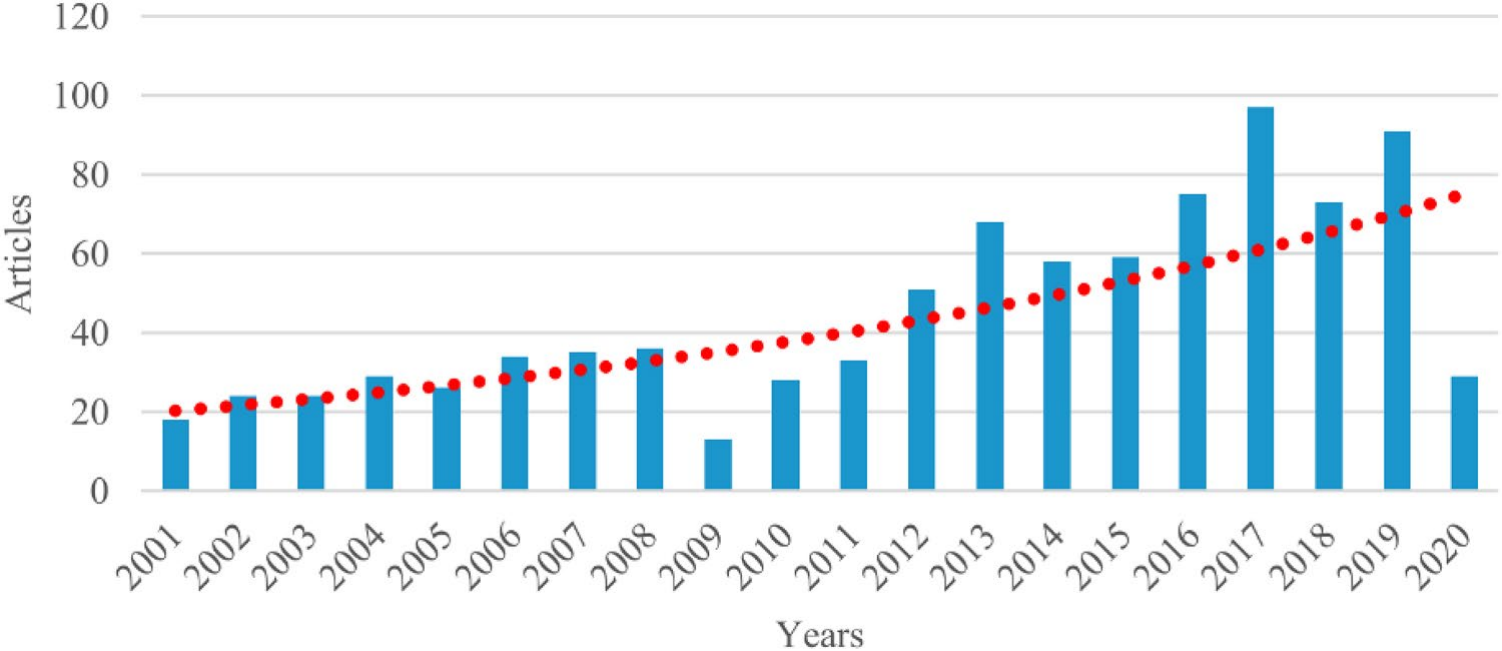
Underwater welding research (Data until the first quarter of 2020)^2^.

Welding under hyperbaric environmental conditions has been used for many years in underwater production and repair. Due to the place of execution, under water, very complex support equipment is sometimes necessary for the execution of the weld seam^1^. A distinction is made between different types of underwater welding processes, for example, welding in dry and wet environments^2^. The equipment for welding in a dry atmosphere can be very complex, similar to a mini habitat for manual welding^1,3^. Furthermore, the research activity around underwater welding has been increasing in the last decade^2^, Fig. 1. The number of published papers has increased and reached approximately 80 expected papers to be published per year in 2020.

One topic in research is the process influence of the water-containing atmosphere and the increased ambient pressure^4^. Furthermore, it was shown that an increased ambient pressure results in a significant reduction in the arc length at the same welding voltage^3,5–9^. In addition to the influence on current and voltage, an influence of the increased pressure on the spatter formation could also be shown^10^.

In the field of materials, a clear influence of the hydrogen content on the microstructure morphology, surface roughness and crack behavior of high-strength steels, which are mostly investigated, was shown^11–15^. Akselsen et al. showed that suitable toughness values for different pressure levels can be achieved using different types of filler metal for X70 pipeline high strength steel^16^. Furthermore, they showed that the penetration depth of the weld is increased due to an increased pressure^16^, and they estimate some practical implications, such as filling root gaps and a reduced layer count. They proposed a first theory for the increased penetration and focused on flow effects in the melt. The stated that the flow is directed downwards to form a deeper penetration due to the elevated pressure.

In addition, the range of materials included in the various investigations is also becoming increasingly broad. For example, the welding of copper or aluminum alloys^17^ and duplex stainless steels^18,19^ under hyperbaric process conditions has been investigated. For aluminum alloys, a beneficial usage of hyperbaric welding in the lower pressure ranges up to 10 bar can be assumed for the reduction of pores, a key problem regarding the welding of aluminum^17^.

For some materials, there is a call for more investigations, such as the welding of molybdenum alloys under hyperbaric process conditions^20^ and for the welding of cladded multimaterial pipelines^21^.

However, a positive benefit in welding production due to an increased ambient pressure has been little addressed in research, but it is of interest since a significant increase in the energy density in the arc can be realized by the arc shortening effect and a corresponding possible voltage increase. In addition, the increased ambient pressure causes the arc to constrict and thus increases the local energy density.

An increased energy density leads to a deeper penetration of the weld seam, as Dutra showed for a modified welding process^22^. Bunaziv et al. showed a similar effect for welding with the CMT mode at elevated ambient pressures^5^.

The increase in the energy density of the arc should result in an increased welding depth and changed solidification conditions, as Azar et al. predicted^23^ and Bunaziv et al. showed for a modified welding process^5^. The arc constriction is, in addition, dependent on the shielding gas mixture used^24^, as for normal GMA welds. Azar modelled and validated the thermal cooling cycles and found an influence of the pressure on the cooling t_8/5_-time but classified this effect as minor^25^. In other studies, the effect on the cooling time can be considered significant. An altered cooling time will lead to different mechanical properties in the weld metal and the HAZ.

The work presented in this paper is intended to shift the focus of hyperbaric welding processes away from underwater welding towards a potential improvement of conventional welding processes. For this purpose, knowledge of the underlying relationships between the welding process boundary conditions, the ambient pressure and the geometric and mechanical weld metal and HAZ (Heat affected zone) properties is crucial. Therefore, in the work presented hereafter, a model is derived for the dependence of the weld geometry on the set variables during hyperbaric welding. The derived statistical model will give an overview of the relationship between the welding depths and weld metal hardness. The focus of the interpretation of the literature and the results in the discussion of the findings will focus on a beneficial use of a hyperbaric process environment in welding without a water-containing setup.

## Experimental setup, materials and methods

**Figure 2:**
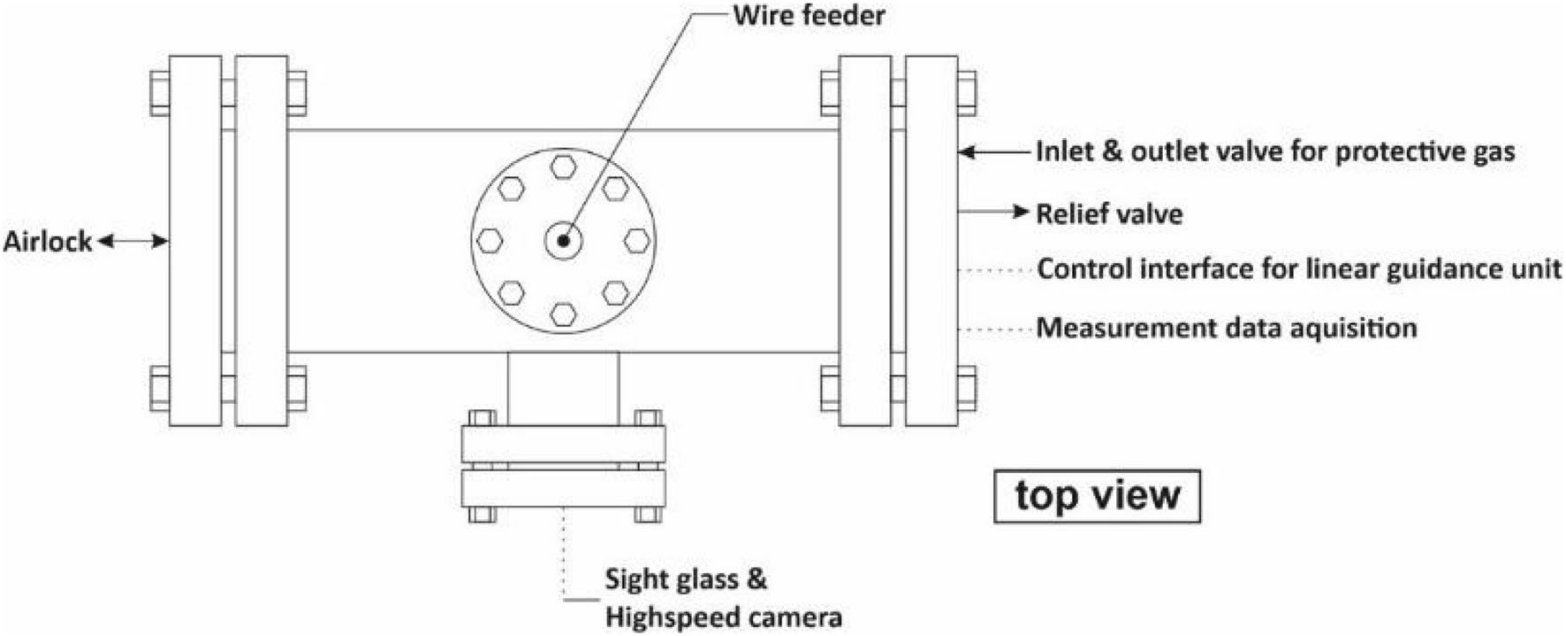
Scheme of the hyperbaric chamber^17^.

**Figure 3:**
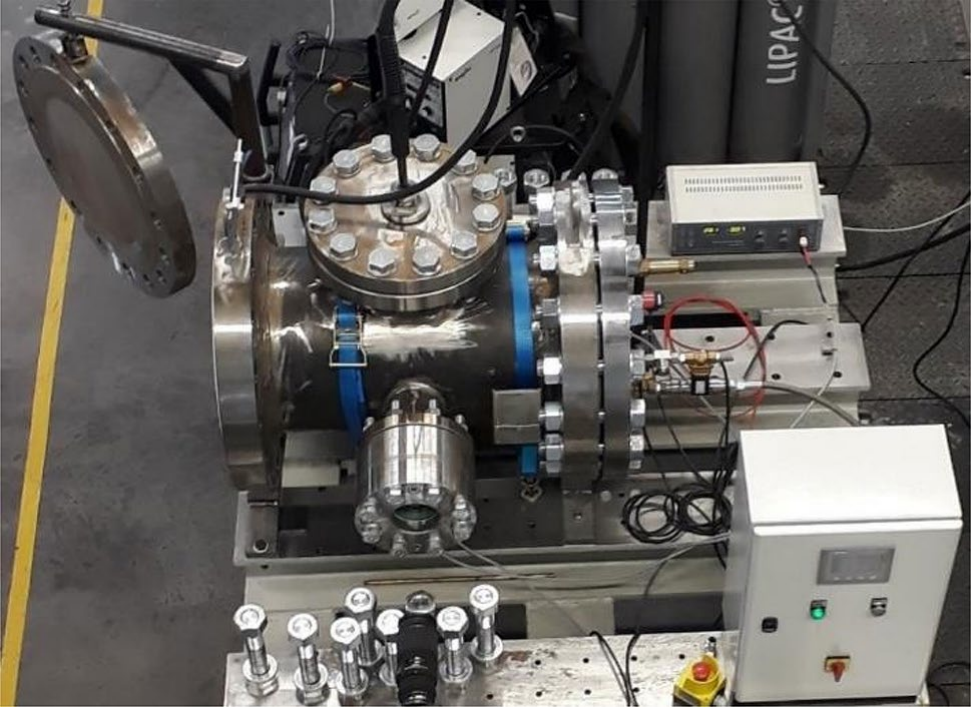
Welding chamber^17^.

To investigate the beneficial use of hyperbaric welding in welding production, a hyperbaric chamber with an external wire feed was realized (Figs. 2 and 3). This chamber can withstand an internal pressure of up to 50 bar and allows visual access to the joining zone. The wire electrode is fed from outside via a gate system. This eliminates the effects of the increased ambient pressure on the welding power source. The sample movement was carried out via a separately controlled linear moving table.

For the following investigations, the ambient pressure of 2 bar to 16 bar, as well as the welding voltage and the welding current, were measured according to a statistical design of experiments in the non-synergy operation mode of the welding power source. Argon was fed into the test chamber as a shielding gas and to top up the pressure to the desired pressure for the experiment.

The chemical composition of the base material used, an S700MC low-alloyed high strength steel, was determined by optical emission spectroscopy. Table 1 shows the results.

**Table 1:** Chemical composition of the base material.

	Contents by Mass in %											
	C	Si	Mn	P	S	Cr	Mo	Ni	Al	Ti	Nb	V
S700MC	0.07	0.26	1.54	0.01	< 0.002	0.05	0.11	0.15	0.036	0.05	0.07	0.07

**Figure 4:**
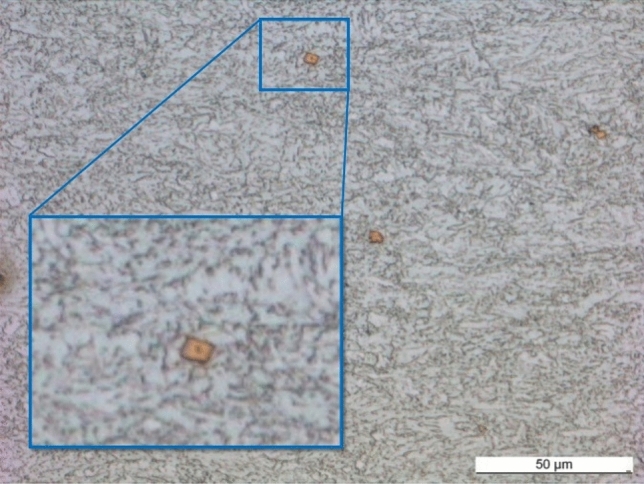
Cross-section S700MC^26^.

As is usual for fine-grained structural steels, microalloys with the elements titanium, vanadium and niobium as well as a larger proportion of manganese are present. The microstructure of S700MC is very fine-grained, ferritic and has a pronounced rolling texture (Fig. 4)^26^.

Larger blocky titanium nitride precipitates (yellowish) are visible throughout the whole microstructure at larger magnifications. Tensile tests were carried out to determine the quasi-static mechanical properties of the test material (Table 2). Therefore, the rolling direction was taken into account while taking the samples^26^. The displayed values are the mean values for the three specimens tested.

**Table 2:** Mechanical properties of the tested material^26^.

	Young’s Module in GPa	Rp0.2 in MPa	Rm in MPa	A in %
S700MC long	187.95	722.0	820.0	10.2
S700MC Transv	202.93	726.0	795.3	12.07

The chemical composition of the weld filler metal was also determined by optical emission spectrometry on two-dimensional build-up welds with 4 layers and is shown in Table 3. The used welding consumable had a diameter of 1.2 mm. The statistical experimental design was set up and evaluated using Modde software (version 12.1).

**Table 3:** Chemical composition of the filler^26^.

Filler	Contents by mass in %								
DIN ISO 16834-A	C	Si	Mn	Cr	Ni	Mo	Cu	Ti	V
G 50 7 M21 4Mo	0.07	0.59	1.56	0.09	0.06	0.45	0.12	0.001	0.008

## Experimental procedure and results

**Figure 5:**
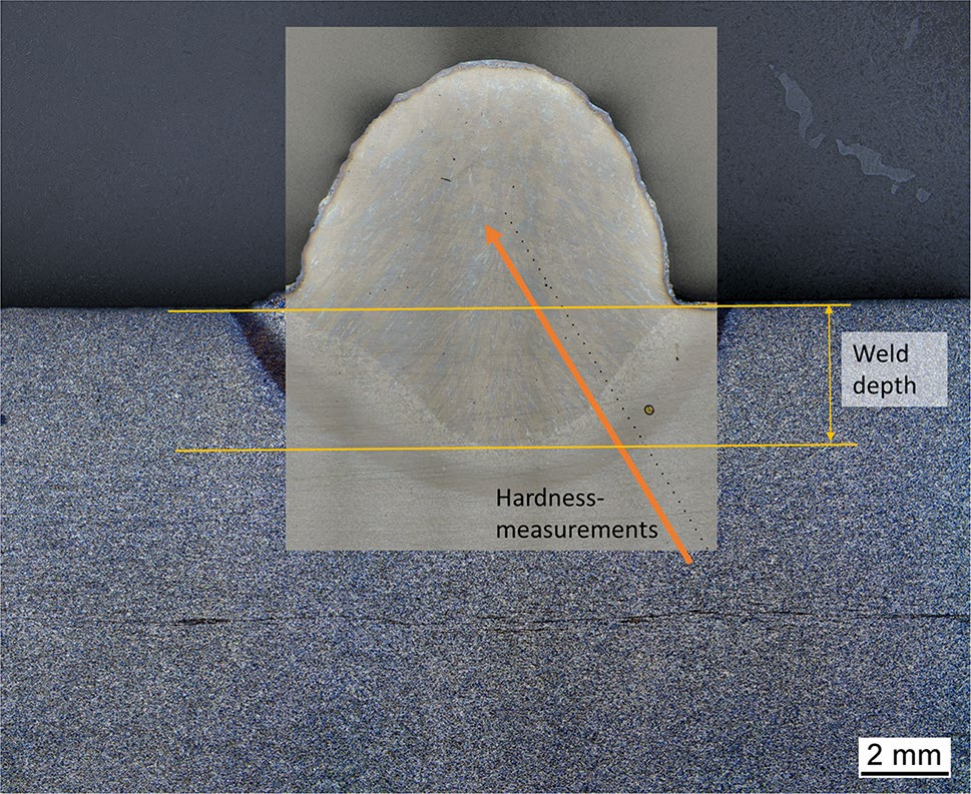
Hardness measurements (example).

The welding tests were carried out following a statistical D-optimal design of experiments (DoE), as shown in Table 4. In the D-optimal experimental design, the determinant of the information matrix is maximized. This criterion leads to the minimization of the volume of the confidence ellipsoid for the unknown parameters of the linear regression model. Furthermore, the DOE provides the opportunity to derive a statistical model to describe the influence of the ambient pressure on various seam characteristics. The changeable variables for the DoE are the welding voltage for a non-synergetic welding process, the ambient pressure, and the welding current. The set point of the wire feed is given as well. Especially the welding current is correlated to the wire feed speed. For the evaluation of the results we choose to use the welding current as setpoint rather than the wire feed speed, because the mean values of the measured currents are corresponding with the set-point values. A significant influence of the ambient pressure on the occurring welding currents and voltages, which has been expected, has not occurred (see section "Cooling times"). Therefore, it si possible, that in this case the welding machine will stick to current as controlled variable. The stick-out was set to 17 mm. The torch was aligned normal, without any angle, to the surface of the used substrate. Table 4 shows the results for the welding depths and hardness (HV0.2) measurements for the bead on plate welding. The hardness measurements (HV0.2) shown in Table 4 are the mean values of the last 10 points in the weld metal of a hardness measurements line starting in the base material, reaching at least into the middle of the weld metal. One example is shown in Fig. 5. The weld depth was determined on a cross-section as shown in Fig. 5.

**Table 4:** Design of experiments with results for weld depth.

Exp No	Wire feed in m/min	Current in A	Voltage in V	Ambient pressure in bar	Weld depth in mm	Hardness in HV0.2
8	9	200	22.5	6.1	2.63	316
1	9	200	26.6	2	3.32	299
9	9	200	26.6667	16	3.63	278
3	9	200	40	2	4.71	253
6	9	200	40	16	5.46	252
10	16.9	350	30	16	7.46	281
16	16.9	350	30	9	5.89	285
15	16.9	350	30	9	6	284
2	23.2	500	30	2	6.09	283
13	23.2	300	22.5	2	6.25	325
14	16.9	350	30	9	6.48	262
12	16.9	350	40	9	8.68	258
17	19	400	40	16	8.87	256
5	23.2	500	35	16	9.31	264
11	23.2	500	30	9	10.2	271
4	23.2	500	40	2	10.42	240
7	23.2	500	40	16	10.53	243

The welding speed was set to 30 cm/min. The welding machine used was an EWM Alpha Q 551. The wire diameter was 1.2 mm, and argon was used as the shielding gas.

**Figure 6:**
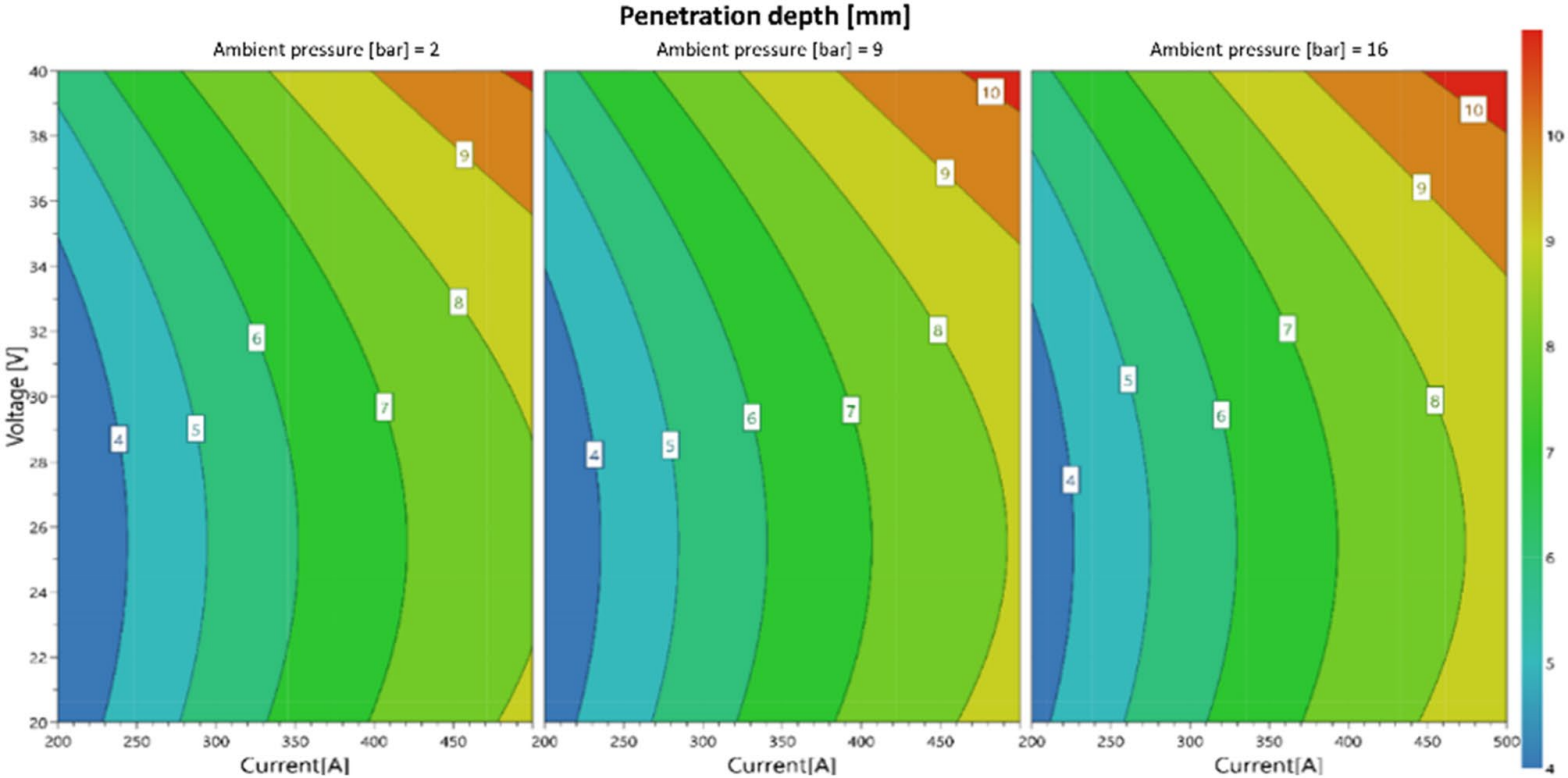
Statistical model for the weld penetration for bead on plate welds.

The derived statistical model is shown in Fig. 6 for the relationship between ambient pressure, welding current, welding voltage and weld penetration depth. The statistical experimental design is shown in Table 4. The influence of the ambient pressure on increasing the welding depth is visible in Fig. 6. As the pressure increases, the welding depth increases. However, the effect is not as pronounced as with the aluminum alloy presented in^17^. These results are supported by findings from Xue et al. presented in^27^. The maximum welding depth is reached at high welding voltages and welding currents.

**Figure 7:**
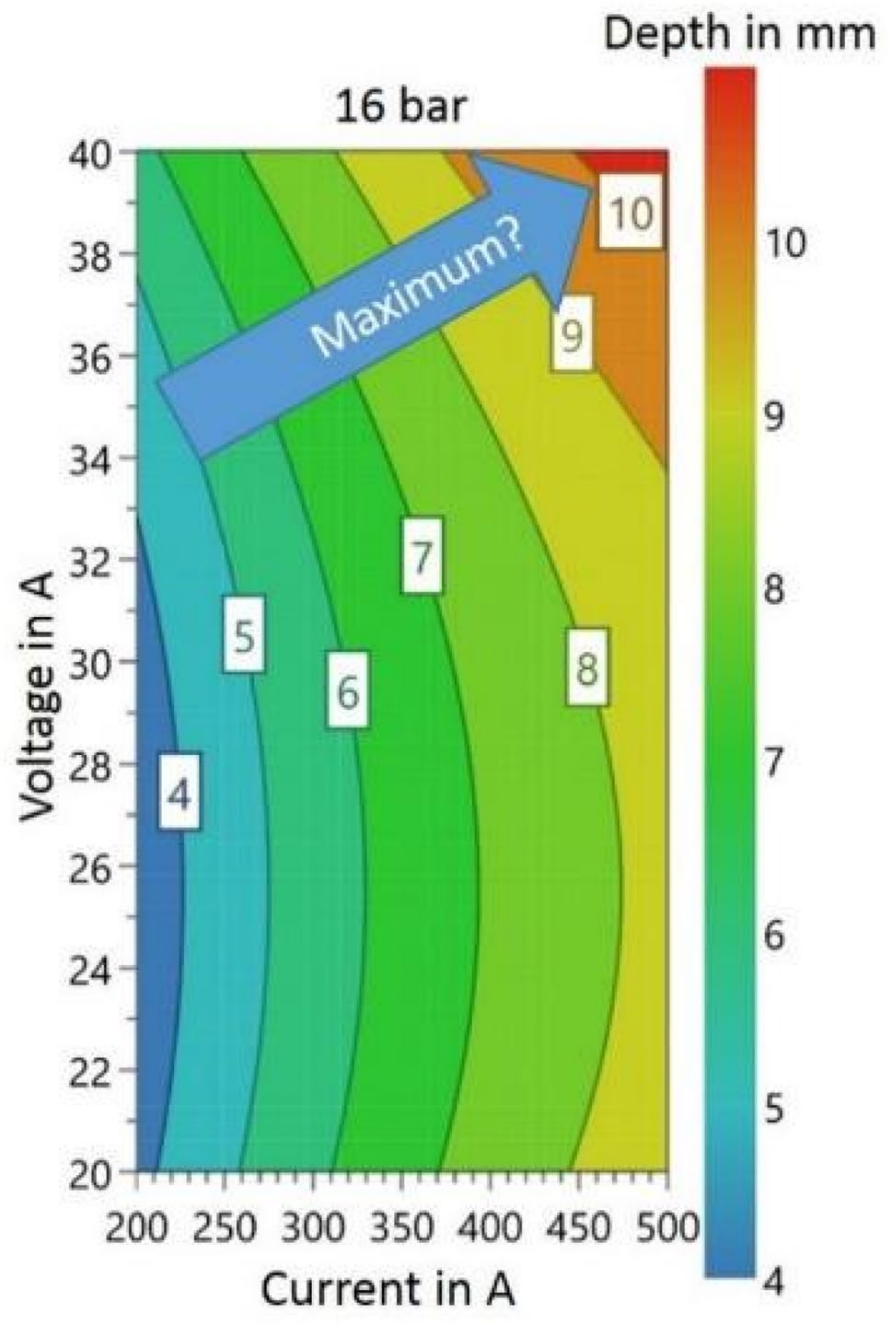
Assumed local maximum in the statistical model.

**Figure 8:**
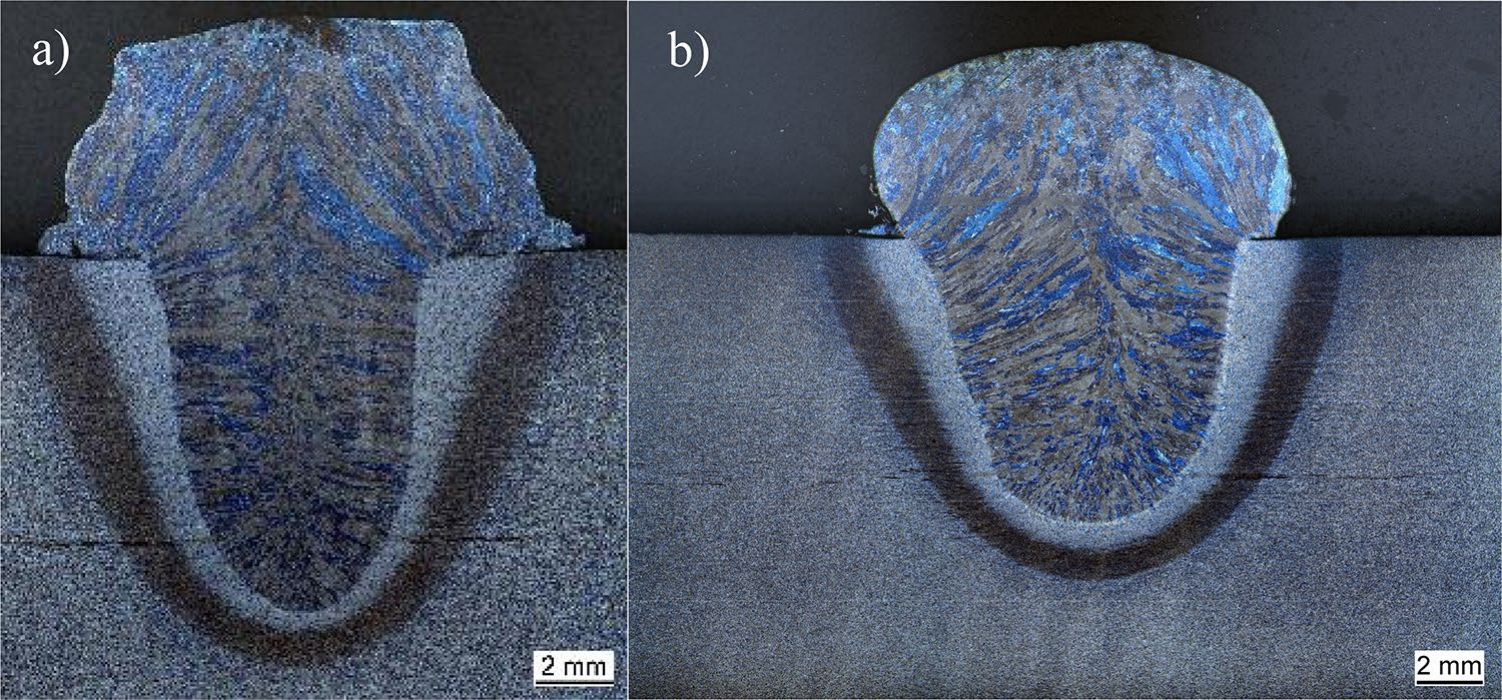
(**a**) Cross-section 500A, 35 V, 16 bar. (**b**) Cross-section 350 A 40 V 9 bar.

These results and findings from studies with aluminum^17,28^, a local maximum for the welding depth as a function of welding voltage and welding current, can be assumed and will form beyond the test area shown, Fig. 7. The welding depth was measured in cross sections taken 50 mm behind the start of the weld. An example of a cross section for the measurement is shown in Fig. 8.

In this case, due to the increased ambient pressure, a more finger-shaped seam geometry in comparison to weld seams at lower pressures occurred, Fig. 8. Furthermore, the observation of the welding processes, the high-energy welding processes with an increased ambient pressure and the finger-like shape in the cross-sections show a buried arc, as Dutra et al.^22^ described in their work. This buried arc has also been seen by visual observation. A buried arc is a welding arc that can occur by welding currents above 500 A^29,30^. This arc burns beneath the surface of the wok piece (Fig. 6, compare^29–32^).

This arc also leads to a more finger-shaped bead geometry and can lead to the usage of smaller angles in weld seam preparation, which can lead to fewer layers that need to be welded for multirun welds^17^. To prove this, some single runs but welds of 15 mm sheets are presented in Sect. “Butt joint” The observed effect of increased energy density in the arc, which in extreme cases leads to a buried arc, in combination with the theory of Akselsen et al.^16^ that the molten pool flow is shaped to produce a deeper penetration should explain the effects described here and in the literature. Baba et al. showed that a buried arc can significantly reduce the number of welding passes required^30^. Due to the constriction of the arc by the increased pressure, the buried arc can also occur at currents below 500 A. The typical downward extended form of the buried arc already occurs at 350 A and a pressure of 8 bar, Fig. 8b.

**Figure 9:**
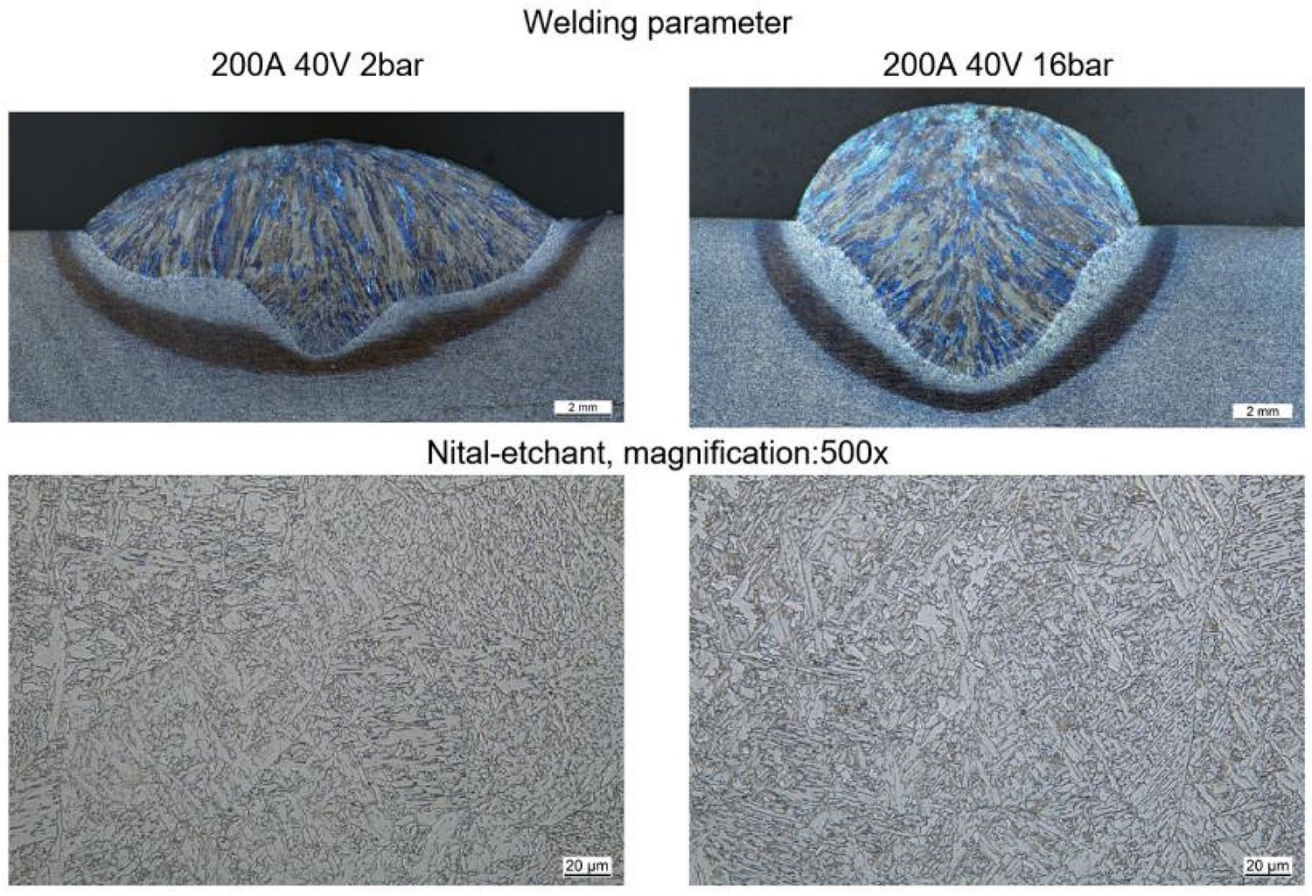
Shape and microstructure of the weld for different ambient pressures.

Figure 9 shows a direct comparison between two weld seams using the same welding voltage and welding current and the same welding speed at different ambient pressures (2 bar and 16 bar). The weld seam under 2 bar is significantly wider than that at 16 bar. On the other hand, the beginning of a finger-shaped weld seam over the entire weld seam width can also be seen at 200 A. As shown in Fig. 9, this finger-shaped feature becomes deeper with increasing welding current. These results show the same tendencies as the literature. In particular, the results of Bunaziv et al. show a significantly increased penetration at elevated pressure^5^.

**Figure 10:**
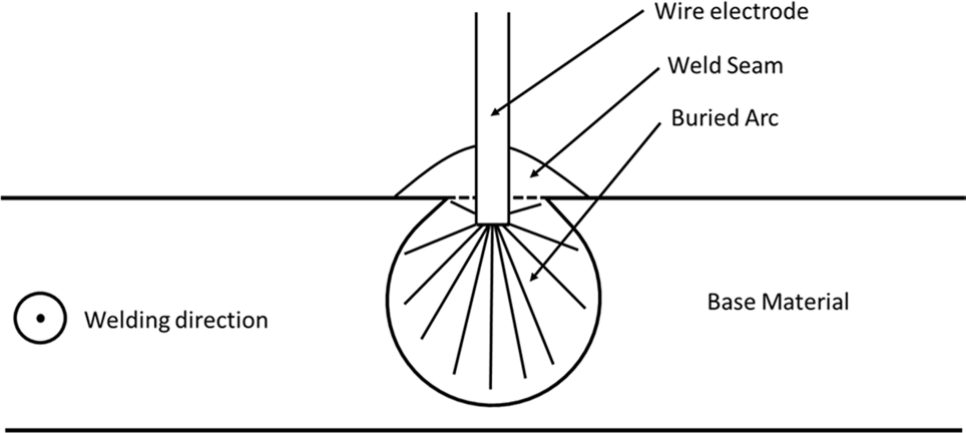
Schematic of a buried arc.

Due to the greater welding depth, shown in Fig. 8, and the occurrence of the buried arc effect, the observation with the high-speed camera leads to inconclusive, nearly black-only pictures. Therefore, the buried arc cannot be presented. In the case of a buried arc, the welding arc burns beneath the base material surface within the material, as shown in Fig. 10.

### Weld metal microstructure

**Figure 11:**
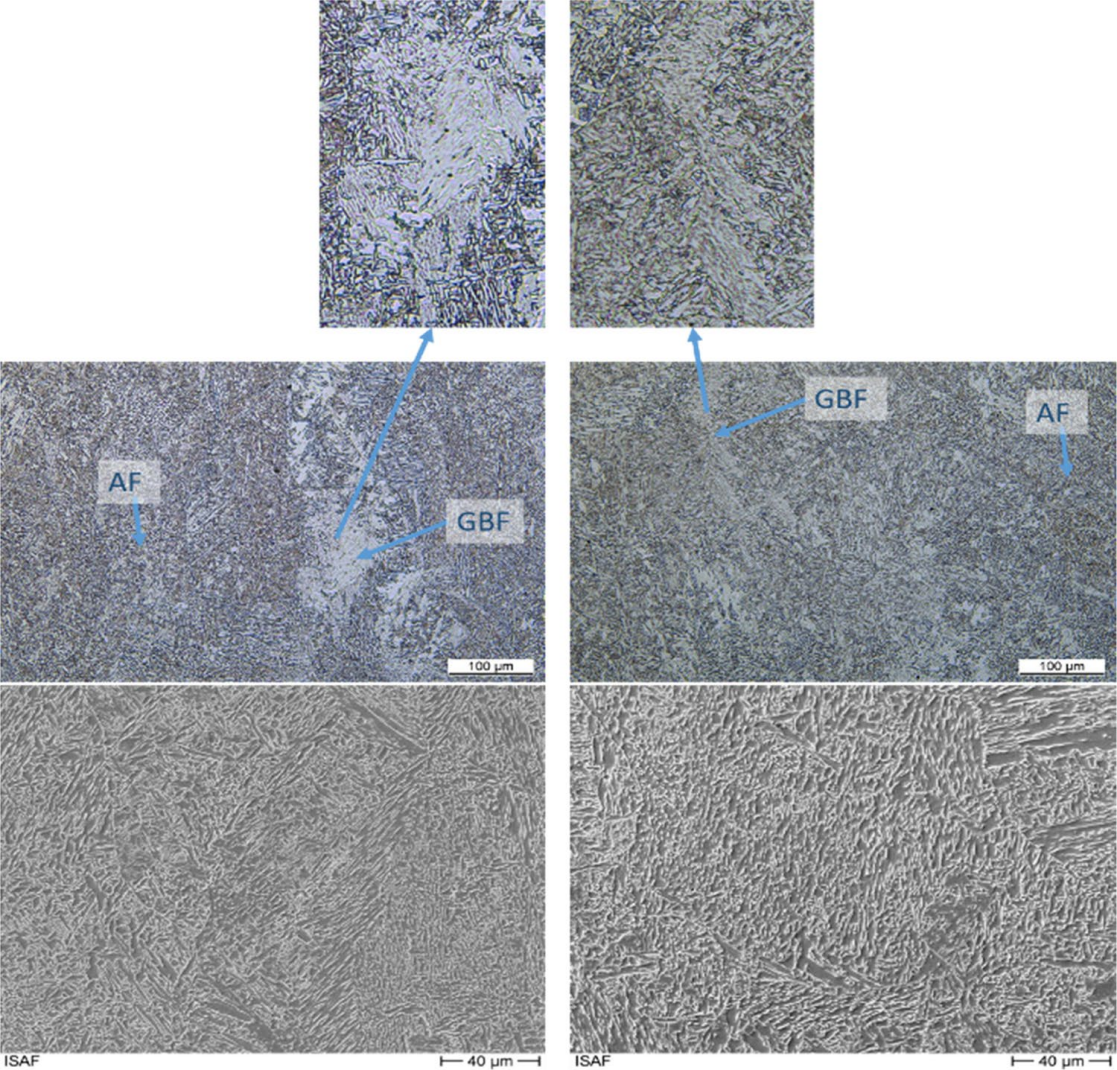
Microstructure. Left side: 500A 30 V 2 bar, right-side: 500A 35 V 9 bar.

The weld seams show a microstructure of acicular ferrite typical for the filler metal used. In addition, grain boundary ferrite is formed for higher joining energy levels. The found microstructure is typical for this type of filler metal^26,33^. For lower energy levels, a slight decrease in grain size can be anticipated, which is also a typical phenomenon for this type of filler. However, a quantitative determination of the grain size of weld seams remains inconclusive. Figure 11 shows the cross section of two low-energy welds under different ambient pressures. In the lower half of the figure, the microstructure of the weld metal can be seen. In both figures, aciucular ferrite can be observed within the former austenite grain boundaries. Furthermore, portions of proeutectoid ferrite are present.

Azar et al. predicted a change in the microstructure for higher welding energy and increased ambient pressure^23^. This is supported by the results. The differences in the microstructure due to different cooling show up for higher energy welding processes, Fig. 11. In this case, in addition to acicular ferrite (AF), grain boundary ferrite (GBF) occurs at low ambient pressure. For the changed cooling due to a higher ambient pressure, the grain boundary ferrite shows a microstructure that indicates faster cooling. This indicates a needle-like bainitic microstructure in the grain boundary ferrite. This is supported by the SEM images of the weld metal centre Fig. 11. Within the grain boundary ferrite, cementite lamellae are increasingly visible.

In summary, it can be said that the microstructure is typical for this filler material and that there are hardly any quantifiable differences between the different energy levels and pressures.

**Figure 12:**
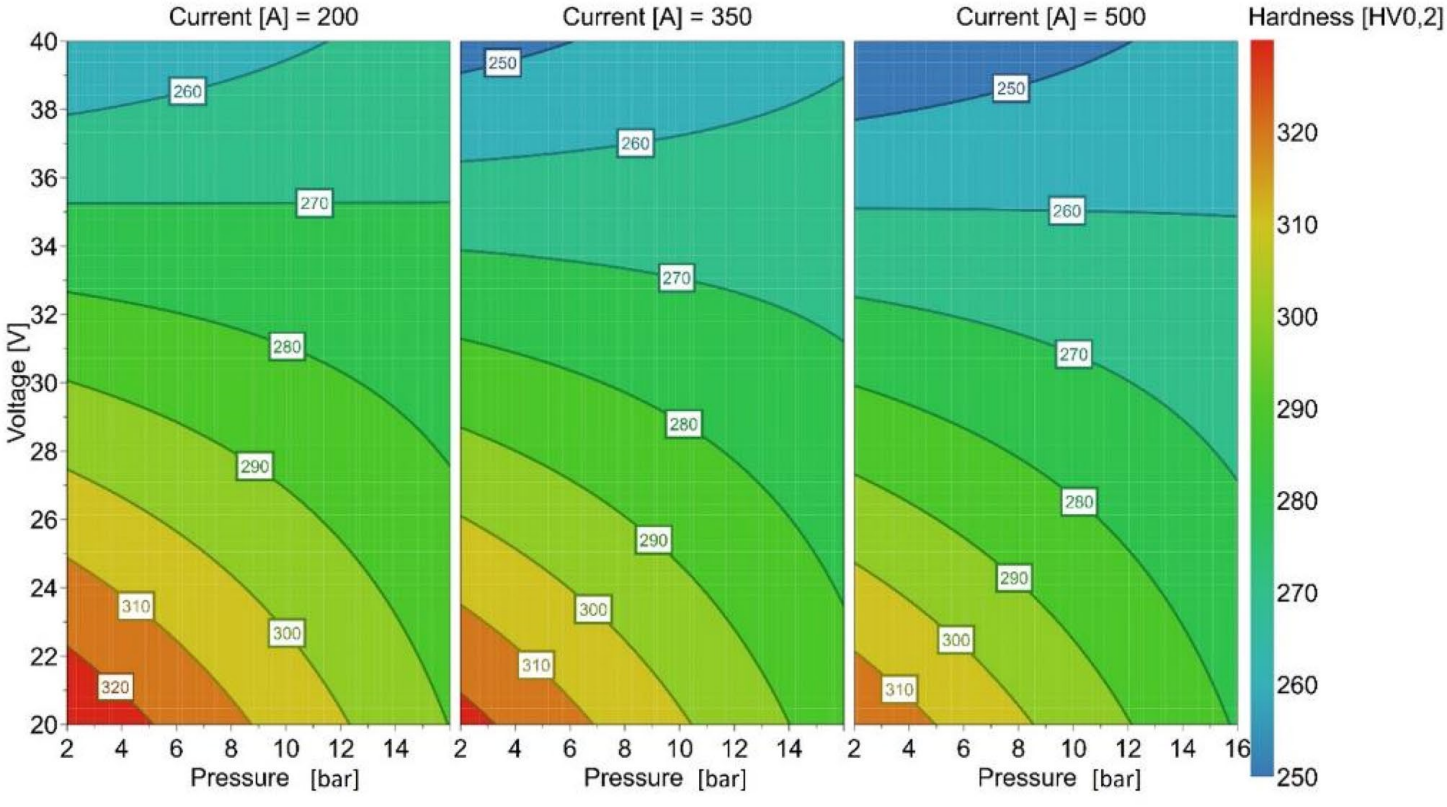
Contour plot hardness in dependence of ambient pressure, welding voltage and welding current.

The results for the characterization of the microstructure coincide with those for the hardness of the welds. To determine the hardness of the weld metal, a microhardness series (HV0.2) was placed from the base metal into the weld metal, with 10 indentations made in the weld metal. The values given in Table 4 are the mean values of the 10 indentations in the weld metal (see Fig. 5). Figure 12 shows the contour plot of the statistical model for hardness as a function of ambient pressure, welding current and welding voltage. With increasing welding current, the hardness decreases, as expected due to the increasing energy per unit length. In addition, Fig. 12 shows that with increasing ambient pressure, there is a slight decrease in hardness in the area of lower welding voltages (below 33 V). At higher welding voltages (above 33 V), there is a slight increase in hardness. For higher welding voltages and higher pressures and/or current, the occurrence of a buried arc lead to a change in the energy dissipation in the material and the welding efficiency will be enhanced. That means, that the weld seam will cool down slower and this leads to lower hardness. If there is now buried arc at lower voltages/currents the arc efficiency well decrease due to the higher amount of gas that will take part in the energy dissipation process. In addition, there is a range at medium welding voltages with no change in hardness due to the ambient pressure. It can be concluded from this that an increase in ambient pressure is likely to change the cooling time. For lower welding voltages, this behaviour is supported by results from Azar et al. and Parshin et al. for higher voltages^25,34^.

### Butt joint

**Figure 13:**
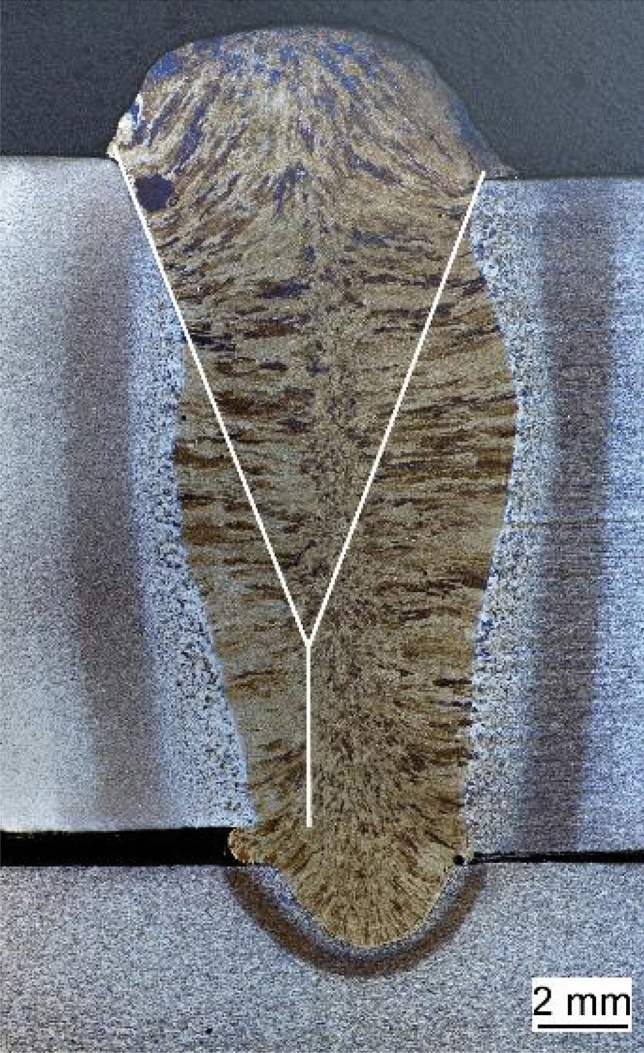
Butt joint 15 mm thickness of base-plate Y-grove weld seam preparation (2 bar).

To demonstrate the positive effects of a hyperbaric process environment for welding fabrication, bars with a sheet thickness of 15 mm made of S700MC were joined with the aid of a backing plate also made of S700MC. To increase the possible weld depth beyond the welding depth selected in the statistical model, a Y-seam preparation with a 5 mm web and an opening angle of 40° was selected. 35 V and 500A (wire feed of 22.3 m/min) were set as process variables. Figure 13 shows a cross-section of the achieved weld at 2 bar. The illustration also shows the weld preparation schematically. The geometry and shape of the welds are comparable at all ambient pressures, and it can be seen that finger-shaped penetration leads to the feasibility of a single-layer 15 mm thick MSG weld even at low ambient pressures. Argon was also used as the shielding gas for these tests. However, there are significant differences in the cooling times. The possibility that a buried arc can contribute to a significant reduction in the number of layers during welding has been demonstrated by Baba et al., among others^30^. Furthermore, they have shown that the necessary weld preparation can also be reduced. The stabilisation of the buried arc at increased pressure shown in the previous chapters demonstrates the potential of the process for welding production, especially for use in the range below 500 A welding current.

#### Cooling times

To determine the cooling times, type-K thermocouples were spotted at a distance of 3 mm to the left and right of the weld seam preparation when welding the butt joints. They were located in the middle of the weld length to be welded.

**Figure 14:**
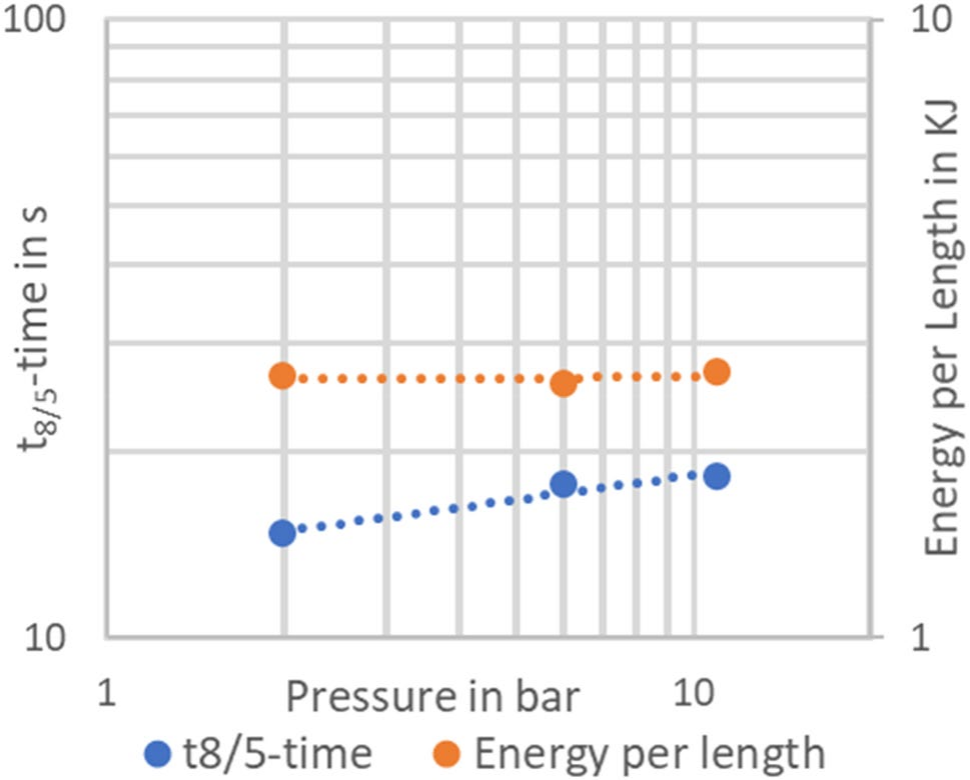
Cooling times and welding energy as a function of the ambient pressure.

Figure 14 shows the dependence of the determined t_8/5_ times on the ambient pressure. Furthermore, the corresponding energy per unit length, which was determined from the average values of welding current and welding voltage, which were also recorded, is shown in Fig. 14. The energy per unit length is not influenced by the ambient pressure. In contrast, the cooling time shows a clear influence of the ambient pressure on the cooling time. Higher ambient pressures lead to an increase in the cooling time, which means that the weld cools down slower. This can be explained by a change in arc efficiency. For higher ambient pressures, the constriction of the arc—which also leads to the buried arc effect—leads to an increase in the arc efficiency. Here, the core point is the change in heat conduction conditions caused by the deeper penetration. This is consistent with the model for the hardness of the weld seam, which shows a slight softening of the weld seam structure, indicating longer cooling times. An influence of an increased pressure on cooling times has been shown by Azar et al.^25^, but their cooling times are 2 s relatively fast, which is typical for underwater applications. In this case, the shown cooling times are in the range between 10 and 20 s, which is typical for “normal” welding of this type of base material. An influence on the arc efficiency was also proposed by Farrell for hyperbaric TIG welding of duplex stainless steels in^19^.

## Summary

The presented work shows that an increased ambient pressure can lead to a deeper and more finger-like weld seam characteristic, which can be used in a non-underwater environment for a reduced angle in weld seam preparation and a reduced layer count due to a deeper penetration of the weld. In addition, the dependency of the arc energy input due to the change in the arc efficiency has been shown, but this needs to be further investigated. The presented results are in line with the existing literature.

The possible reduction of weld seams and reduction of the weld seam preparation have been exemplarily shown for a single seam joint of a 15 mm thick plate with a 40° Y-shaped weld seam preparation with 5 mm.

Furthermore, statistical models were set up for the hardness and penetration as a function of the process values. The derived statistical model can help to identify the needed process values of the welding process, including the ambient pressure for a targeted welding depth per layer.

By stabilising the buried arc through the increased ambient pressure, the number of welding layers required for thick-walled components can be significantly reduced. However, further development steps are necessary for the implementation of hyperbaric welding on thick-walled components. Nevertheless, it could also be shown that the pressures required to stabilise the buried arc are relatively low and that it should therefore be possible to implement a concept for locally increasing the pressure without a chamber.

## Outlook

In addition to the presented investigations, the use of a mixture of 82% argon and 18% carbon dioxide as the shielding gas is planned. The determination of the influence of ambient pressure on toughness and other mechanical properties is also pending. In this case a detailed study of the HAZs microstructure is planned as well. Furthermore, the next large step is the development of an out-of-chamber hyperbaric welding torch to utilize the effects shown.

## Data Availability

The datasets used and/or analysed during the current study are available from the corresponding author on reasonable request.

## References

[1] Alajmi EF, Alqenaei AA (2017). Underwater welding techniques. IJERA.

[2] Surojo E, Syah Putri EDW, Budiana EP (2020). Recent developments on underwater welding of metallic material. Procedia Struct. Integr..

[3] Ofem, U. U. Laser assisted arc welding process for dry hyperbaric deep water application, Cranfield University.

[4] Parshin SG, Levchenko AM, Maystro AS (2020). Metallurgical model of diffusible hydrogen and non-metallic slag inclusions in underwater wet welding of high-strength steel. Metals.

[5] Bunaziv I, Aune R, Olden V (2019). Dry hyperbaric welding of HSLA steel up to 35 bar ambient pressure with CMT arc mode. Int. J. Adv. Manuf. Technol..

[6] Fydrych D, Kozak T (2009). Underwater welded joint properties investigation. Adv. Mater. Sci..

[7] Gyasi, E. A. Welding processes of metals for offshore environment: underwater welding. Lappeenranta-Lahti University of Technology LUT (2019).

[8] Łabanowski J, Fydrych D, Rogalski G (2008). Underwater welding: a review. Adv. Mater. Sci..

[9] Tang, D., Niu, H., Xue, L. *et al.* Study on underwater hyperbaric dry GMAW welding. in *Proceedings of the 2017 7th International Conference on Manufacturing Science and Engineering (ICMSE 2017)*. Atlantis Press, Paris, France (2017).

[10] Li K, Gao H, Li H (2014). Droplet rebounded spatter in dry hyperbaric gas metal arc welding process. Int. J. Adv. Manuf. Technol..

[11] Chen H, Guo N, Liu C (2020). Insight into hydrostatic pressure effects on diffusible hydrogen content in wet welding joints using in-situ X-ray imaging method. Int. J. Hydrogen Energy.

[12] Klett J, Hecht-Linowitzki V, Grünzel O (2020). Effect of the water depth on the hydrogen content in SMAW wet welded joints. SN Appl. Sci..

[13] Kong X, Li C, Zou Y (2016). Measurement and analysis of the diffusible hydrogen in underwater wet welding joint. MATEC Web Conf..

[14] Tomków J, Fydrych D, Rogalski G (2019). Efecto del sistema de apantallamiento de la soldadura y el tiempo de almacenaje de los electrodos en el contenido de hidrógeno difundido en el metal depositado. REVMETAL.

[15] Gheonea MC, Florescu SN, Mihailescu D (2020). Influence of marine corrosion on the roughness of the dry hyperbaric underwater MAG welding joints. IOP Conf. Ser. Mater. Sci. Eng..

[16] Akselsen, O. M., Hårsvæ, A., Fostervoll, H. *et al.* Root bead profiles in hyperbaric GTAW of X70 pipeline. *Int. J. Offshore Polar Eng.* **16**(2), S123–127 (2006).

[17] Treutler K, Brechelt S, Wiche H (2021). Beneficial use of hyperbaric process conditions for welding of aluminium and copper alloys. Weld. World.

[18] Hu Y, Shi Y-H, Shen X-Q (2017). Microstructure, pitting corrosion resistance and impact toughness of duplex stainless steel underwater dry hyperbaric flux-cored arc welds. Materials (Basel).

[19] Farrell, J. Hyperbaric welding of duplex stainless steel pipelines offshore, Cranfield University.

[20] Zhu Q, Xie M, Shang X (2020). Research status and progress of welding technologies for molybdenum and molybdenum alloys. Metals.

[21] Bunaziv I, Olden V, Akselsen OM (2019). Metallurgical aspects in the welding of clad pipelines—a global outlook. Appl. Sci..

[22] Dutra JC, Gonçalves e Silva RH, Riffel KC (2020). High-performance GMAW process for deep penetration applications. Weld. World.

[23] Azar, A. S., Fostervoll, H., Akselsen, O. M. Prediction of the thermal cycles in dry hyperbaric GMA welding using partial differential heat transfer equations. in *Conference: 9th International Conference on Trends in Welding Research American Society for Metals*.

[24] Azar AS, Ås SK, Akselsen OM (2013). Analytical modeling of weld bead shape in dry hyperbaric GMAW using Ar-He chamber gas mixtures. J. Mater. Eng. Perform..

[25] Azar, A. S., Akselsen, O. M., Fostervoll, H. Prediction of the thermal cycles in dry hyperbaric GMA welding using partial differential heat transfer equations (2012).

[26] Treutler, K. Schweißen von Leichtbaurahmenkonstruktionen: funktionale Werkstoffauswahl und Schweißzusatzwerkstoffmodifikation, Universitätsbibliothek Der TU Clausthal (2019).

[27] Xue L, Wu J, Huang J (2016). Welding polarity effects on weld spatters and bead geometry of hyperbaric dry GMAW. Chin. J. Mech. Eng..

[28] Brechelt, S., Wiche, H., Treutler, K. *et al*. Hyperbares schweißen von aluminiumlegierungen. in *40. Assistentenseminar Fügetechnik: DVS Berichte, Band: 357, 1. Auflage 2019*, vol. 357. DVS Media GmbH, Düsseldorf (2019).

[29] Perić M, Garašić I, Tonković Z (2019). Numerical prediction and experimental validation of temperature and residual stress distributions in buried-arc welded thick plates. Int. J. Energy Res..

[30] Baba H, Era T, Ueyama T (2017). Single pass full penetration joining for heavy plate steel using high current GMA process. Weld. World.

[31] Baba H, Honda R, Era T (2020). Microstructure observation of high-current buried-arc welded joint. Q. J. Japan Weld. Soc..

[32] Dreveck NW, Barbetta LD, Bond D (2020). Influence of push and pull techniques on high-speed buried-arc GMAW process. Soldag. Insp..

[33] Treutler K, Wesling V (2020). Usage of Ti-surface-modified filler material to increase the joint strength of high-strength low alloyed (HSLA) steels under different load types. SN Appl. Sci..

[34] Parshin S, Levchenko A (2020). Underwater hyperbaric dry welding of high strength steel arctic oil and gas pipelines. IOP Conf. Ser. Earth Environ. Sci..

